# Ten-year experience of an outpatient clinic for CKD-5 patients with multidisciplinary team and educational support

**DOI:** 10.1007/s11255-021-02963-y

**Published:** 2021-07-31

**Authors:** Vincenzo Terlizzi, Massimo Sandrini, Valerio Vizzardi, Mattia Tonoli, Annalisa Facchini, Luigi Manili, Letizia Zeni, Giovanni Cancarini

**Affiliations:** 1 Operative Unit of Nephrology, ASST Spedali Civili Brescia, Piazzale Spedali Civili, 1, 25123 Brescia, Italy; 2 Postgraduate School in Nephrology, University of Brescia, Brescia, Italy

**Keywords:** Start of dialysis, Patient survival, GFR trajectory, Delaying dialysis, Optimal start of dialysis, End-stage renal disease

## Abstract

**Purpose:**

To analyze the results of an outpatient clinic with a multidisciplinary team and educational support for patients with late-stage CKD (lsCKD), to check its possible effect on their outcomes.

**Methods:**

Longitudinal cohort study on patients followed up in the MaReA (Malattia Renale Avanzata = CKD5) outpatient clinic at ASST Spedali Civili of Brescia from 2005 to 2015 for at least six months. Trajectory of renal function over time has been evaluated only in those patients with at least four estimations of eGFR before referring to MaReA.

**Results:**

Seven hundred and six patients were enrolled, their mean age was 72 ± 14 years, 59% were males. At the end of the study, 147 (21%) were still on MaReA, 240 (34%) on dialysis, 92 (13%) on very low-protein diet (VLPDs), 13 (2%) on pre-hemodialysis clinic, 23 (3%) improved renal function, 10 (1%) transplanted, 62 (9%) transferred/lost to follow-up, and 119 (17%) died. Optimal dialysis start (defined as start with definitive dialysis access, as an out-patient and without lsCKD complications) occurred in 180/240 (75%) patients. The results showed a slower eGFR decrease during MaReA follow-up compared to previous renal follow-up: − 2.0 vs. − 4.0 mL/min/1.73 m^2^ BSA/year (*p* < 0.05), corresponding to a median delay of 17.7 months in dialysis start in reference to our policy in starting dialysis. The patient cumulative survival was 75% after 24 months and 25% after 70. Limitations: (1) lack of a control group, (2) one-center-study, (3) about all patients were Caucasians.

**Conclusion:**

The follow-up of lsCKD patients on MaReA is associated with an optimal and delayed initiation of dialysis.

## Introduction

The incidence and prevalence of chronic kidney disease (CKD) are progressively increasing worldwide [1]. In Italy, three studies on the prevalence of CKD show different results: CKD 3–5 stages account for 6% for patients aged 18–95 years in the GUBBIO Study[2], CKD1–4 12.3% in people aged over than 40 in the INCIPE Study [3] and 6.3% in the CARHES Study on 35–79-year-old patients [4].

Although stage 5 represents the most advanced stage of CKD, it is still possible at this stage to reduce the prevalence and severity of end-stage kidney disease-related complications and postpone the need of dialysis [5–7]. Two factors could play impact positively on CKD outcomes: (a) early referral to nephrology care [8–10], (b) outpatient clinic evaluation by a multidisciplinary team, with exposure to educational program and cumulative “dose” of nephrological care. Notably, several works have recently been devoted to these topics [7, 11–15].

The present longitudinal cohort study analyzes a 10-year experience in outpatient clinic dedicated to the End-Stage Kidney Disease ESKD (in Italian: Malattia Renale Avanzata, MaReA) with a multidisciplinary team and educational support.

## Patients and methods

The MaReA outpatient clinic was born at the Nephrology Operative Unit—ASST Spedali Civili and University of Brescia, in February 2005. Essentially, MaReA includes CKD patients with eGFR < 15 mL/min/1.73 m^2^ BSA; however, few patients with several and severe comorbidities have been accepted even with slightly higher values.

The inclusion criteria were: (1) first access to MaReA from 1 February 2005 to 31 August 2015, (2) follow-up at MaReA over a period of time equal or greater than 6 months. For the calculation of changes in trajectory of renal function, a minimum of four estimations on eGFR (by MDRD or CKD-EPI) during the 6 months prior to entry in MaReA were required; this choice was done to ensure more accuracy and reliability in calculating linear regression coefficient. Follow-up ended at the time of last evaluation in MaReA.

MaReA organization is based on a multidisciplinary approach, including different professional healthcare providers as nephrologists, nurses, dieticians, and social workers with the aim to provide patients with a holistic perspective of their chronic condition. The educational support was provided by the nephrologists, at each bimonthly visit, by nurses with 2–4-h meetings and by dieticians for a total of 1.5–2.0-h. At the time of clinical evaluation, patients were screened for any possible lsCKD complications (e.g.: badly controlled blood pressure, nausea, vomiting, hyperkaliemia, fluid overload, metabolic acidosis, malnutrition) and new onset of extra-renal/cardiovascular comorbidities was checked. Changes of pharmacological and dietary therapy, as well as in lifestyle were suggested at the end of clinical evaluation. In case a bimonthly visit was considered insufficient, the subsequent clinical evaluation was scheduled on a tight timeline. Patients were offered the opportunity of an individual meeting with a nurse to receive exhaustive information on principal CKD-related complications, renal replacement therapy strategies and appropriate diet and lifestyle and therefore reinforce and further explain concepts introduced during medical evaluation. From a clinical point of view, attention was focused on self-assessment of hydration status. In addition to medical evaluation and meeting with nurses, patients were referred to a dietitian for a low-protein and low-salt diet (0.6–0.8 g proteins/kg BW; and 35 kcal/kg/day in subjects aged < 60 years and 30 kcal/kg/day in those > 60 years) [16, 17] with minimization of potassium and/or phosphate dietary intake and appropriate intake of high biological value proteins from vegetal instead of animal sources.

The diagnostic and therapeutic approach to lsCKD complications has changed and updated consistently to new international/national guidelines procedures on CKD5 published over time (see Appendix 1).

The study was conducted in accordance with the Helsinki Declaration. The data were recorded anonymously and included, clinical information (demography, primary renal disease, type and time of dialysis access, comorbidities, hospitalizations, complications, and outcome) and biochemical parameters: hemoglobin, albumin (Alb), calcium (Ca), phosphate (P), parathyroid hormone (PTH).

The patients were divided into three groups based on the duration of nephrological care: group 1 with less than six visits in MaReA; group 2, from six to ten visits; group 3 more than ten visits. Patients were also classified according to the mode of dialysis start: optimal vs. non-optimal, with ‘optimal’ meaning start with definitive dialysis access (i.e.: distal or proximal arterio-venous fistula for HD patients and peritoneal catheter for patients starting on peritoneal dialysis), as an out-patient and in the absence of any acute lsCKD-related complications; non-optimal start was defined as absence of one out of three ‘optimal’ conditions.

Prior to MaReA follow-up, few patients did not exhibit at least four eGFR estimates (by both MDRD and CKD-EPI formulas). Among those with at least four eGFR estimates before MaReA, the trajectory of renal function (RF) over time was analyzed by means of least squares according to many works recently published [6–9]. The regression line of the pre-MaReA eGFR trajectory has been extrapolated to predict the hypothetical time to start dialysis; the estimate of the delay in starting dialysis was calculated by subtracting this time to the real time when dialysis was started in those patients.

Since the beginning of MaReA practice all clinical and biochemical data were recorded in a FileMaker^®^ file (Apple Inc.; California, USA) and subsequently retrieved in Excel^®^ (Microsoft Office, Microsoft Corporation, Seattle, WA, USA) and eventually analyzed with Statgraphics^®^ (Statpoint Technologies Inc.; Virginia, USA).

Data are reported as mean plus/minus standard deviation or median and interquartile range (IQR), as appropriate. The Chi-square test was used to compare categorical variables, Student’s t test for continuous variables with normal distribution, and Kruskal–Wallis to compare medians. Univariate survival analysis was performed by the Kaplan–Meier method and the curves were compared with the log-rank test. Multivariate survival analysis was performed using the Cox’s proportional risk method.

A *p* value of 0.05 was accepted as statistically significant.

## Results

### Population characteristics

**Fig. 1:**
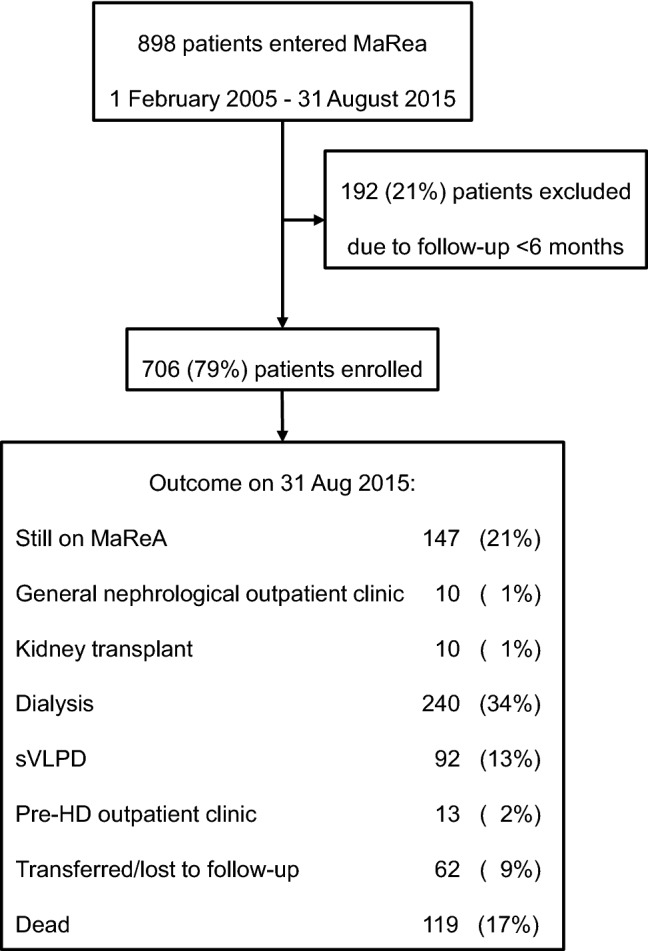
Flow chart of patient selection and outcome

During the period starting from 1 February 2005 to 31 August 2015, 898 patients accessed to MaReA; 706 of them (79%) met inclusion criteria and were enrolled to the study. 414 were males (59%), with an average age of 72 ± 14 years (range: 14–98), BMI 26 ± 5 kg/m^2^ and median follow-up in MaReA 348 days (IQR: 208; 711). Figure 1 shows the flow chart of patient’s selection and enrollment. The main characteristics of study population are summarized in Table 1. During the follow-up period, 240 patients started dialysis.

**Table 1:** Characteristics of the population studied

Number of patients	706
Male gender	414 (59%)
Age (years)	72 ± 14
Ethnicity	
Caucasian	691 (98%)
African	10 (1%)
Asian	5 (1%)
Primary renal diseases	
Unknown/missing	288 (41%)
Glomerulonephritis	106 (15%)
Diabetic	101 (14%)
Vascular	98 (14%)
Tubular-Interstitial	50 (7%)
ADPKD	40 (6%)
Other	23 (3%)
Comorbidities	
Hypertension	638 (90%)
Diabetes mellitus	282 (40%)
Ischemic heart disease	268 (38%)
Cardiac arrhythmia	258 (37%)
Previous diagnosis of malignancy	228 (32%)
Peripheral vasculopathy	216 (31%)
Cerebral vasculopathy	215 (30%)
Urinary tract infection	179 (25%)
Dyslipidemia	160 (23%)
Chronic respiratory disease	147 (21%)
Cirrhosis or chronic liver disease	101 (14%)

### Laboratory tests

Table 2 summarizes of the laboratory tests performed during the follow-up period. Hemoglobin values (Hb) at the end of the study were statistically lower compared to the beginning (*p* < 0.05). Over time, a statistically significant increase was found in *p* (*p* < 0.05) and PTH (*p* < 0.05) levels; no significant changes occurred in Ca (*p* = 0.88) and Alb (*p* = 0.74).

**Table 2:** Laboratory test values during the follow-up

	Entry	6th month	12th month	EOF	*p*
Hemoglobin (g/dL)	11.6 ± 1.4	11.7 ± 1.5	11.6 ± 1.3	11.2 ± 1.4	< 0.05
serum Albumin (g/dL)	3.8 ± 0.6	3.9 ± 0.5	4.0 ± 0.5	3.8 ± 0.5	NS (0.74)
serum Calcium (mg/dL)	9.1 ± 0.8	9.2 ± 0.7	9.2 ± 0.6	9.1 ± 0.8	NS (0.88)
serum Phosphate (mg/dL)	4.1 ± 0.9	4.1 ± 0.8	4.0 ± 0.8	4.4 ± 1.1	< 0.05
PTH (pg/mL);	208 (128; 336)	219 (139; 317)	213 (134; 329)	228 (131; 394)	< 0.05

Statistical comparison has been done between entry and end of follow-up (EOF): Data reported as M ± SD for Hb, serum Albumin, Calcium and Phosphate, and as median and IQR for PTH

### Outcome and start of dialysis

At the end of the study, 147 (21%) patients were still in MaReA follow-up, 240 (34%) started dialysis, 92 (13%) were shifted to a very low-protein diet supplemented with amino acids (VLPDs) 13 (2%) to pre-hemodialysis clinic (out-patient clinic for patients with already functioning vascular access for hemodialysis, 23 (3%) to general nephrological outpatient clinic due to partial RF recovery, 10 (1%) received a kidney transplant, 62 (9%) were moved to different hospitals / lost to follow-up and 119 (17%) died. Among patients who started dialysis, 167 (70%) chose hemodialysis and 73 (30%) peritoneal dialysis without statistically significant difference among the three groups of duration of pre-dialysis nephrological care. The main indications for start dialysis were inadequate electrolyte/fluid control and trend toward malnutrition.

The optimal start of dialysis occurred in 180 patients (75%) without statistically significant difference among the 3 groups of duration of care (Table 3).

**Table 3:** “Optimal” dialysis initiation in the 240 patients who started dialysis and comparison of the three groups of patients divided according to the duration of nephrological care given

Mode of starting dialysis	Tot	%	Group 1 < 6 visits in MaReA	Group 2 6–10 visits in MaReA	Group 3 > 10 visits in MaReA	*p*
(1) with definitive access	201	84%	104 (82%)	55 (86%)	42 (84%)	0.83
(2) without complications	228	95%	120 (95%)	62 (97%)	46 (92%)	0.49
(3) as outpatient	203	85%	103 (82%)	56 (88%)	44 (88%)	0.44
Optimal start (1 + 2 + 3)	180	75%	91 (72%)	50 (78%)	39 (78%)	0.58

### eGFR change over time

**Fig. 2:**
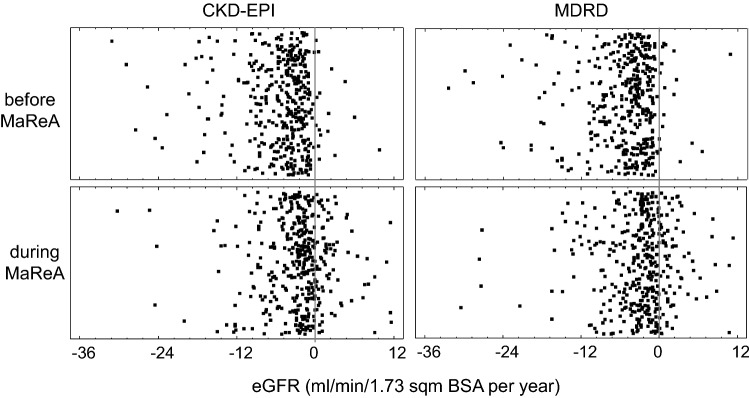
Changes in eGFR with CKD-EPI and MDRD formula before and during MaReA. Seven outliers in each panel have not been shown to increase readability of the figure

**Fig. 3:**
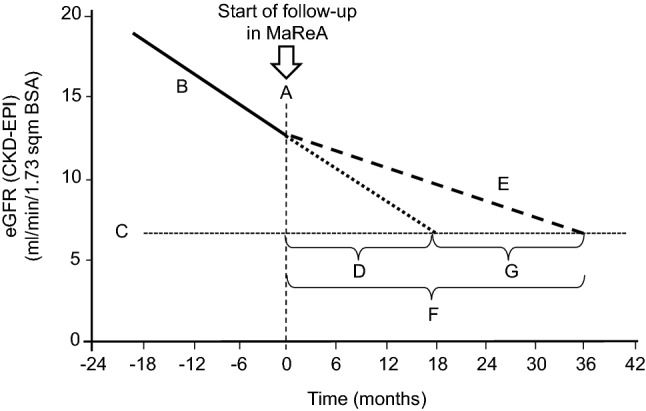
Median eGFR decrease according to CKD-EPI equation. Continuous line: eGFR decrease in pre-MaReA period; dotted line: expected trajectory of eGFR after starting MaReA, according to the previous decrease rate; dashed line: observed eGFR decrease during MaReA period. See Table 4 for further explanation

Progression of renal dysfunction over time has been studied only in those 396 patients (56%) who had at least four values of eGFR before entering MaReA. The worsening of eGFR-CKD-EPI rate was significantly higher before the patients entered MaReA than during the follow-up in MaReA: − 4.0 (− 7.1; − 2.2) mL/min/1.73 m^2^ BSA/year vs. − 2.0 (− 4.9; − 0.3) mL/min/1.73 m^2^ BSA/year; (*p* < 0.05); similar results were detected for the eGFR by the MDRD equation (*p* < 0.05) (Table 4). The single points of the changes are reported in Fig. 2. The reduction in the rate of worsening of RF can be extrapolated to a median postponement in the start of dialysis of 17.7 (1.5; 30.3) (eGFR by CKD-EPI) months or 16.0 (1.6; 29.5) months (eGFR by MDRD) (Fig. 3).

**Table 4:** Median and IQR of eGFR values and progression rate (see also Fig. 1)

	CKD-EPI	MDRD	Reference in Fig. 1
Median eGFR when entering in MaReA (mL/min/1.73 m^2^ BSA)	12.6 (10.2; 16.0)	14.2 (11.4; 17.9)	**A**
Annualized decrease in eGFR before entering in MaReA (mL/min/1.73 m^2^ BSA/year)	− 4.0 (− 7.1; − 2.2)	− 4.0 (− 7.1; − 2.2)	**B**
Median of observed eGFR at start of dialysis (mL/min/1.73 m^2^ BSA)	6.7 (5.3; 8.5)	7.7 (6.1; 9.4)	**C**
Expected lag time between entry in MaReA and start of dialysis according to extrapolation of eGFR trajectory observed before entering MaReA (months)	17.7 (12.3; 21.9)	19.5 (14.4; 24.3)	**D**
Annualized decrease in eGFR while in MaReA (mL/min/1.73 m^2^ BSA/ year)	− 2.0 (− 4.9; − 0.3)	− 2.2 (− 4.7; − 0.3)	**E**
Expected lag time from entry in MaReA to start of dialysis according to extrapolation of eGFR trajectory observed during MaReA (months)	35.4 (24.6; 43.8)	35.5 (26.2; 44.2)	**F**
Difference in months between real and expected start of dialysis (months)	17.7 (1.5; 30.3)	16.0 (1.6; 29.5)	**G**

### Hospitalization

Patients with optimal start of dialysis were hospitalized, during dialysis period, 1.4 times/year vs. 2.1 times/year of patients with non-optimal start (*p* < 0.001); the median days of hospitalization were, 8 (IQR 2–23) per year vs. 20 (IQR 11–57) (*p* < 0.001), respectively. CKD complications occurred more frequently in patients with not-optimal start of dialysis (*p* < 0.001).

No significant differences in the rate of hospitalizations were noticed between the three groups of duration of pre-dialysis nephrological care.

### Survival

Among the 706 patients enrolled in the study, 119 (17%) died during the follow-up. MaReA patients had 75% cumulative survival (Kaplan–Meier curve) after 24 months, 50% after 41 and 25% after 70 months, respectively.

Patients aged over 75, had a median survival lower than those younger than 75: 2.82 vs. 4.68 years (*p* < 0.001) at univariate *as treated* analysis. A significant difference in patient survival was not found neither among the three groups of different duration of nephrological care (*p* = 0.327) nor between optimal vs. non-optimal start of dialysis comparison (*p* = 0.380).

Cumulative patient survival after starting dialysis was 75% after 2.9 years and 50% after 8.5 years.

Cox’s analysis showed that the only covariates that significantly affect survival were age at the start of dialysis, diabetes, COPD (Chronic Obstructive Pulmonary Disease) and chronic liver disease (Table 5).

**Table 5:** Cox analysis for risk of death in dialysis

	HR	CI 95%	*p*
Age at start of dialysis (years)	1.099	1.066–1.133	< 0.001
Diabetes	1.816	1.127–2.329	0.014
Chronic obstructive pulmonary disease	1.691	1.019–2.806	0.042
Chronic liver disease/cirrhosis	1.711	1.050–2.787	0.031

## Discussion

The high prevalence of CKD [1–4], the late referral to nephrologist and the chronic need of dialysis reduce the quality of life of patients and affect negatively healthcare costs.

In this context, nephrologists should make an effort to reduce CKD progression and delay the need of start of dialysis. Importantly, one of the mainstays for an accurate and effective management for a chronic condition as CKD is patient educational program. The educational program consists of a multilevel and multidisciplinary approach to provide patient with awareness of his/her disease, related complications, and possible therapeutic options.

The present study reports the results of a 10-year field experience in an outpatient clinic (MaReA) dedicated to CKD5 patients. The studied population was old (72 ± 14 year) and affected by many co-morbidities (Table 2): almost all patients showed hypertension (90%), 40% suffered from diabetes mellitus; 38% had ischemic heart disease and 32% had a history of malignancy. The high proportion of patients with unknown etiology of renal disease could depend partly on late referrals and also to our policy of define the etiology of underlined nephropathy only when proved by renal biopsy, sonography imaging, and immunological laboratory test. The high mean age and a median of four comorbidities per patient highlight the frailty of study population.

The laboratory results were satisfactory throughout the follow-up period; hemoglobin was 11.6 ± 1.4 g/dL when patients entered MaReA and showed a statistically significant reduction to 11.2 ± 1.4 g/dL (*p* < 0.05) only at the end of follow-up. However, anemia was barely controlled with Hb values being over the minimum value recommended by KDIGO guidelines [10]. Serum albumin remained stable at3.9 g/dL during the follow-up, these data support the efficacy of care in maintaining an adequate nutritional status also in lsCKD [18]. Mineral bone disease control met the recommended targets [19] although a moderate increase in serum phosphate and PTH was observed.

During the follow-up, 240 patients (34%) started renal replacement treatment; peritoneal dialysis accounted for 30%, the figure is above the average of 26% of Italian PD Centers in 2014 [20] and the 18% of our Region Lombardy [21]. These data suggest that that incidence of DP is higher when patients are adequately informed and involved in the choice of dialysis modality [22]. To some extent, time spent in MaReA could have increased knowledge of the disease and helped patients to become more self-confident of home treatment. However, changes in patient self-confidence in home dialysis were not assessed; thus, it remains a mere speculation. The duration of nephrology care did not significantly influence the penetration of peritoneal dialysis in our study, probably because even in patients with a shorter MaReA follow-up, the time was sufficient to provide with adequate information for the choice of dialysis modality.

The optimal start of dialysis is of paramount importance, as demonstrated by its association with a lower mortality rate at 6 months [23]. An optimal start of dialysis occurred in the majority of patients (75%). In a Canadian series, the corresponding figure was only 40% of the whole population of 339 patients and mainly initiating with a temporary vascular access [23]. The use of temporary central venous catheters (CVC) is associated with increased inflammatory status and higher mortality [24–26]. CVC were used in 80% of American patients starting dialysis [27] and in 40% of incidents dialysis patients in Italy [28]. In our study, a large proportion of patients (84%) of patients started hemodialysis with a definitive vascular access. In addition, 85% of our patients started dialysis as outpatient. The start as out-patient reduces the risks associated with hospitalization and the cost for the National Health Service (NHS). Singhal et al. reported than 60% of patients with a follow-up of at least 12 months in a pre-dialysis outpatient clinic, started dialysis as out-patients [7]. In 8856 patients from two National French registry, the lack of follow-up with a nephrologist was associated with the start dialysis in emergency [12].

According to the European Renal Best Practice [29], the policy of our Operative Unit of Nephrology is starting dialysis before patients become symptomatic for CKD complications. Obviously, to avoid uremic complications in patients with eGFR between 6 and 9 mL/min/1.73 m^2^ BSA, a multilevel and personalized clinical surveillance should be offered. A large number of patients (95%) started dialysis in the absence of acute uremic complications. An earlier start of dialysis was excluded since median eGFR-CKD-EPI was 6.7 (IQR 5.3; 8.5) mL/min/1.73 m^2^ BSA. Although the most appropriate value of eGFR to start dialysis has not been established yet, the Ideal study, the only randomized controlled trial on this topic, did not show differences in mortality between patients with “early-onset” of dialysis (10–15 mL/min/1.73 m^2^ BSA) and those ones with “late” start (5–7 mL/min/1.73 m^2^ BSA) [30].

Many reports suggest that eGFR decrease over time has a linear decline in the majority of CKD5 patients [31] with a worsening rate of 2.2–6.4 mL/min/1.73 m^2^ BSA [2, 7–11]. In our study, the worsening rate of eGFR (CKD-EPI) slowed down from − 4.0 mL/min/1.73 m^2^ BSA/year before MaReA to − 2.0 mL/min/1.73 m^2^ BSA/year during MaReA (*p* < 0.05). A reduced worsening rate in eGFR has been reported in patients referred to a multidisciplinary outpatient clinic when compared to patients on “usual care” (− 5.1 vs − 7.3 mL/min/1.73 m^2^ BSA/year) [33].

This improvement in the trajectory of RF is associated with a delay in the start of dialysis of 17.7 (CKD-EPI) or 16.0 months (MDRD). Postponing dialysis initiation could reduce the number of complications related to dialysis and vascular access, could improve patient quality of life and reduces healthcare costs for NHS. In Italy, the average annual cost for a CKD5 patient in pre-dialysis is about € 5229 [34] much lower than the 26,797 per year on dialysis [35].

Uremic patients are burdened with a high rate of hospitalization. In our series, the median of hospitalization was 1.6 admissions/patient-year equivalent to about 12 days/patient-year, greater than data reported by other centers in Italy [36, 37]. In our series, optimal dialysis start was associated with a 33% reduction in number of admissions and 60% of days of hospitalization. The reduction was associated to reduction in hospitalizations due to uremic complications, whereas there was no difference for the other possible causes of admission. These data support the hypothesis that the MaReA clinic could offer good clinical control as suggested by some works supporting that a multidisciplinary approach is the keystone to reduce hospitalizations [33, 38–40].

At the end of the observation, 21% of patients were still in MaReA while 17% were died. Median cumulative survival was 40.9 months. The study of Chandna et al^.^ showed a lower median survival (21.2 months vs 40.9 months) in CKD5 patients, but they were older than our patients (77 vs. 72 years)[41].

The cumulative patient survival after starting dialysis was 75% after 2.9 years and 50% after 8.5 years; the 2016 ERA-EDTA Registry reports a lower, 45%, 5-year patient survival [42]. This difference could be an effect of the multi-disciplinary clinical–educational approach, as suggested by other papers [43–45].

The patient survival in dialysis showed no significant differences between the groups with different duration of care as well as between optimal vs. non-optimal start of dialysis; one possible explanation is that the whole population was followed for at least 6 months in MaReA and therefore all of them benefited from a careful management of pre-dialysis complications.

Current literature on conservative, multidisciplinary and educational management of lsCKD is lacking. In the 440 CKD patients reported by Awdishu et al. [11], only 17 were on stage 5 CKD.

Our study has some limitations: the single-center nature, very limited ethnical variability and the absence of a control group. In addition, the hypothetical start time of dialysis is not an actual datum, but an extrapolation based on the changes in eGFR trajectory. On the other hand, some papers support that lower eGFR decrease is strongly associated with the subsequent development of established end points and suggest the possibility of using eGFR decline as a surrogate end point [46–48].

The results of this study suggest that a multidisciplinary team, an educational support and a bimonthly frequency of medical visits could guide patients through the stage 5 CKD safely and is associated with a good nutritional status and an optimal and delayed start of dialysis. Probably, also the earliest stage of renal disease could benefit from multidisciplinary and educational approach, in terms of postponement /avoidance of RRT.

## Data Availability

Data not available, due to their sensitive nature.

## Appendix 1

Updated Guidelines and Renal Best Practice used as reference for the lsCKD outpatient clinic. Previous version versions of some guidelines are now not available.

1. **Società italiana di nefrologia**: Best practice and Procedures.
  Websites: https://bestpractice.sinitaly.org/; http://www.nephromeet.com/web/eventi/NEPHROMEET/index.cfm; https://documenti.sinitaly.org/linee-guida-e-best-practice/; https://documenti.sinitaly.org/malattia-renale-cronica/; http://www.salute.gov.it/portale/documentazione/p6_2_2_1.jsp?lingua=italiano&id=2244&id=2244
  http://www.nephromeet.com/web/lib/Download.cfm?dirdownload=E%3A%5Cgruppotesi%5Cdatasite%5Cnephromeet%5CDocs%2FDOCSIN%5CATT%5C&filename=58%5FLG%5FMRC2012%2Epdf&filesavename=LG%5FMRC2012%2Epdf&typeattach=inline
2. **Italian ministry of health**: Sistema Nazionale della Linee Guida dell’Istituto Superiore di Sanità. Websites: https://www.iss.it/linee-guida1; https://snlg.iss.it/?cat=59
3. **European renal association—European Dialysis Transplantation Association** (ERA-EDTA): European renal best practice on CKD. Website: https://www.era-edta.org/en/erbp/guidance/chronic-kidney-disease/position-statements-and-endorsements/
4. **Kidney Disease: Improving global outcomes** (**KDIGO)**. Website: https://kdigo.org/guidelines/
5. **National Kidney Foundation Kidney Disease Outcomes Quality Initiative (NKF-KDOQI)**. Website: https://www.kidney.org/professionals/guidelines
6. **Diagnosis, therapy and care procedures of the ASST Spedali Civili di Brescia.** Some of them are common to many hospital wards and some are dedicated only to the Operative Unit of Nephrology. Only for internal use; not available on Internet.

## Code availability

Data recorded on FileMaker^®^ file (Apple Inc.; California, USA) and retrieved in Excel^®^ (Microsoft Office, Microsoft Corporation, Seattle, WA, USA).
